# CoNSEnsX: an ensemble view of protein structures and NMR-derived experimental data

**DOI:** 10.1186/1472-6807-10-39

**Published:** 2010-10-29

**Authors:** Annamária F Ángyán, Balázs Szappanos, András Perczel, Zoltán Gáspári

**Affiliations:** 1 Laboratory of Structural Chemistry and Biology, Institute of Chemistry, Eötvös Loránd University, Budapest, Hungary; 2 ELTE-HAS Protein Modeling Group, Budapest, Hungary; 3 Evolutionary Systems Biology Group, Institute of Biochemistry, Biological Research Center, Szeged, Hungary

## Abstract

**Background:**

In conjunction with the recognition of the functional role of internal dynamics of proteins at various timescales, there is an emerging use of dynamic structural ensembles instead of individual conformers. These ensembles are usually substantially more diverse than conventional NMR ensembles and eliminate the expectation that a single conformer should fulfill all NMR parameters originating from 10^16 ^- 10^17 ^molecules in the sample tube. Thus, the accuracy of dynamic conformational ensembles should be evaluated differently to that of single conformers.

**Results:**

We constructed the web application CoNSEnsX (Consistency of NMR-derived Structural Ensembles with eXperimental data) allowing fast, simple and convenient assessment of the correspondence of the ensemble as a whole with diverse independent NMR parameters available. We have chosen different ensembles of three proteins, human ubiquitin, a small protease inhibitor and a disordered subunit of cGMP phosphodiesterase 5/6 for detailed evaluation and demonstration of the capabilities of the CoNSEnsX approach.

**Conclusions:**

Our results present a new conceptual method for the evaluation of dynamic conformational ensembles resulting from NMR structure determination. The designed CoNSEnsX approach gives a complete evaluation of these ensembles and is freely available as a web service at http://consensx.chem.elte.hu.

## Background

Protein NMR is the method of choice for determining protein structures at the atomic level in solution. In addition, NMR experiments allow characterization of protein dynamics at a wide range of time scales [1-7]. Dynamical studies of the past decade led to the emerging paradigm that the so-called 'native structure' of a protein can be better viewed as a number of more or less similar conformers interconverting on different time scales. Functional interactions perturb this state by shifting the equilibrium towards 'active conformations' that are present but are low-populated in the apo state. The most extreme examples of this kind of behavior are provided by intrinsically disordered proteins (IDPs) that adopt a plethora of diverse conformations in their free state but, at least some of them, might become fully or partially well ordered upon partner molecule binding [8,9].

IDPs can not be described with single-conformer models but only with conformational ensembles capturing the diversity of structures. Nevertheless, even the conformational heterogeneity of globular proteins due to their internal dynamics requires the use of such representations. In turn, these can be useful to understand details of molecular interactions and function [10]. The so-called dynamic conformational ensembles reflecting the flexibility of proteins can be regarded as a novel type of models of protein structure. It should be kept in mind that all representations of protein structures are actually models of the 'real' ones and thus can have different types of errors. Precision comes from experimental uncertainty, whereas accuracy reflects the correspondence to reality [11]. Accuracy can only be reliably assessed by means of independent measurements which can range from obtaining distinct parameter sets not used for structure calculations (cross-validation) to the reproduction of the full structure determination procedure by a different research group.

The use of dynamic structural ensembles is further supported by a notion put forward recently on the example of H-D exchange protection factors, namely that it is not reasonable to assume that even a single molecule exists in the NMR tube fulfilling all measured NMR parameters simultaneously [12]. Thus, both the generation and evaluation of dynamic structural ensembles is based on treating NMR observables as ensemble properties, instead of stemming from a single conformer (for review, see e.g. [13]).

Currently there are a number of methods to treat several types of NMR-derived restraints as ensemble properties during structure refinement, such as NOEs [14-16], S^2 ^values [17], RDCs [18] and CSA values [10,18]. It should be noted that different types of restraints are effectively averaged over different ensemble sizes, a problem addressed by the MUMO (minimal under-restraining minimal over-restraining) approach [15]. Protocols aimed at generating ensembles reflecting the internal dynamics of proteins include DER (dynamic ensemble refinement [14]), MUMO [15] and EROS (ensemble refinement with orientational restraints [10]). It should be mentioned that NMR-derived information can be also used in a time- (rather than ensemble-) averaged manner [18-20] and that other types of restraints are increasingly used for the determination of heterogeneous structural ensembles [13,21,22].

Structural ensembles that reflect NMR-derived parameters better than 'conventional' ones are not necessarily derived from simulations restrained with these data. For example, multiple X-ray structures may reflect differences occurring in solution [23], or the existing variability in conformer sets can be extracted and complemented for more complete sampling of structural heterogeneity [24]. Other ensemble-generating approaches, such as inferential structure determination (ISD for short [25,26]) avoiding the inherent errors in conventional single-conformer refinement methods have also been put forward.

Although there are programs (e.g. Xplor-NIH [27]) allowing ensemble refinement of a number of NMR parameters, to our knowledge, there are currently no approaches incorporating all measurable NMR-derived parameters in structure calculations in an ensemble-averaged manner. One of the reasons of this is clearly the growing number of such parameters. However, as it was shown recently, this might not be even necessary, as dynamic protein ensembles generally reproduce even parameters not used for their calculations better than conformer sets obtained with single-structure refinement [14,15]. Thus these parameters can be used as independent factors for the cross-validation of structural ensembles.

In this paper we report the development and evaluation of the CoNSEnsX approach (Consistency of NMR-derived Structural Ensembles with eXperimental data), capable of comparing NMR-derived parameters with the corresponding ones back-calculated from a protein structural ensemble. The method is available as a web service and is aimed at promoting the generation of dynamic conformational ensembles and their use in understanding the links between protein dynamics and function. To demonstrate the capabilities of the CoNSEnsX method and the features of dynamic structural ensembles, we present a detailed analysis of different ensembles of three proteins: human ubiquitin as a well-characterized and relatively rigid protein, a 35-residue protease inhibitor as a small flexible protein and a disordered protein.

## Results

### The CoNSEnsX web server

In protein NMR, the widely used structure calculation protocols, termed single conformer refinement (SCR) methods below, yield a family of conformers, each and every one aimed at corresponding to a set of experimental restraints as much as possible. This also means that despite early expectations, these conformer ensembles are not necessarily suitable to analyze the internal dynamics of the molecules. Therefore, the calculation of dynamic structural ensembles is a separate task yielding conformer sets that might substantially differ from SCR-derived ones [28].

**Figure 1:**
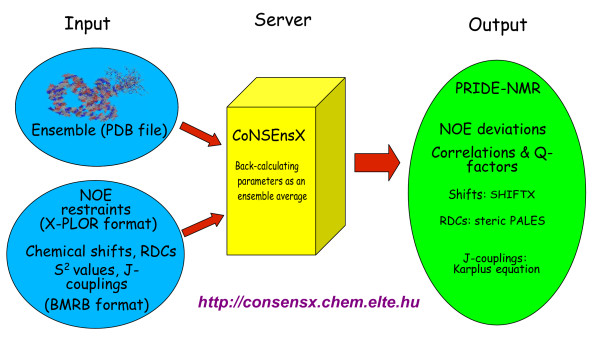
**Scheme of the CoNSEnsX approach**. Gives the schematic structure of the CoNSEnsX web server.

Motivated by the ensemble view of protein structures and aimed at providing standardized tools for the analysis of dynamically relevant structural ensembles of proteins, we developed an application, CoNSEnsX (Consistency of NMR-derived Structural Ensembles with eXperimental data) capable of evaluating the correspondence of NMR-derived parameters to structural ensembles as a whole (Figure 1). The justification for our approach is that ensemble averaging is a key component of CoNSEnsX which would need extra, although relatively simple, calculations even for programs that could be run separately, like SHIFTX [29] and PALES [30]. CoNSEnsX is designed to offer unbiased and ready-for-use structural ensemble evaluation.

The CoNSEnsX program is designed as an easy-to-use tool taking three files as input, a PDB formatted file containing the atomic coordinates of the protein conformers, an X-PLOR/CNS formatted distance restraints file and an NMR-STAR file containing all available NMR parameters the user wishes to evaluate (Figure 1). We note that there are no required parameter types as the program skips the missing ones (it is not expected that all the parameters manageable by the program are indeed available for a given system; see also later).

The program is capable of evaluating the following types of experimental parameters against those back-calculated from the ensemble:

• ^1^H-^1^H distance restraints are evaluated twofold:

◦ PRIDE-NMR, assessing the correspondence of each conformer to the set of NOE restraints [31].

◦ Ensemble-averaged restraint violations (optionally).

• S^2 ^order parameters.

• Chemical shifts (using the SHIFTX program [29]).

• Residual dipolar couplings (RDC) (using the PALES program [30]).

• Several types of scalar couplings that can be back-calculated using the φ backbone dihedral.

The CoNSEnsX web server is freely available at http://consensx.chem.elte.hu.

We note that NOE violation calculation is different from that implemented in standard quality-checking tools and thus their results are not directly comparable to those obtained using CoNSEnsX. For full details of the calculations, see Methods.

For S^2 ^order parameters, chemical shifts and couplings, CoNSEnsX returns the correlation between experimental and back-calculated values, an ensemble Q-factor and an RMSD. Also, a histogram with the distribution of the PRIDE-NMR values per structural model and another with NOE restraint violations is returned. We have refrained from combining the results of CoNSEnsX into a single measure of quality for several reasons. First, CoNSEnsX by no means replaces commonly used structure validation tools such as PROCHECK-NMR [32], which serve a different purpose. Second, the type and amount of NMR parameters available for different structures varies greatly, rendering a single quality measure meaningless for comparing different ensembles evaluated with different sets of parameters.

The server also returns a diagram depicting the relationship between the correlation of experimental vs. back-calculated values obtained for individual structures and the full ensemble. This yields information about whether the use of ensemble representation can be justified on the basis of better reproducing the experimental values for that particular parameter type.

### Analysis of structural ensembles of human ubiquitin

We chose human ubiquitin as the first test protein for the evaluation of the CoNSEnsX approach. Human ubiquitin is probably the most thoroughly studied protein by NMR spectroscopic methods and a wide range of structures determined with different methods and under different conditions are available. Also, there are a number of different experimental data sets available for ubiquitin, making it an ideal first test candidate for CoNSEnsX. Moreover, it can be characterized by high backbone Lipari-Szabo S^2 ^parameters indicating a fairly rigid structure at the ps-ns time scale.

We have used as many as 16 different structural ensembles of ubiquitin, taken from publicly available databases such as the PDB [33] and the RECOORD [34], and we have generated three additional structural ensembles specifically for this study. The various ubiquitin ensembles used for evaluating their correspondence to experimental data are summarized in

**Figure 2:**
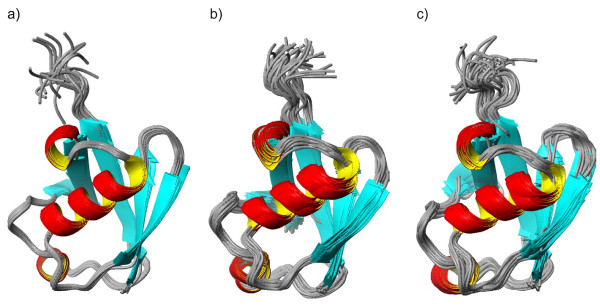
**Ribbon representation of human ubiquitin ensembles generated for this study**. A: U_COCO ensemble (20 members); B: U_NNR ensemble (32 conformers); C: U_1UBQMD ensemble (32 conformers). Figures were prepared with MOLMOL.

Table 1[10,14,15,26,34-38]. The list contains the X-ray structure of ubiquitin and a number of NMR-derived structures, including dynamic conformational ensembles determined recently. The publicly available ISD (inferential structure determination [26]) ensemble is also used. Besides these, we have generated three additional ensembles: one with the COCO (complementary coordinates [24]) method capable of complementing ensembles with additional conformers to reflect the full diversity observed in the original ensemble ('U_COCO' ensemble, Figure 2A), and two derived from molecular dynamics simulations, one restrained using experimental data (termed 'U_NNR' for NOE+NH S^2^+RDC data used for its calculation; Figure 2B) and one unrestrained (termed '1UBQ_MD' for the X-ray structure used as a starting conformer; Figure 2C). Before submission to the CoNSEnsX server, all ensembles were superimposed to the backbone of all residues with the program MOLMOL [39].

**Table 1:** Human ubiquitin ensembles used for evaluation with the CoNSEnsX approach.

Structure identifier	Description	No. of models	Reference(s)
U_1D3Z	Solution NMR ensemble	10	[35]

U_COCO	Solution NMR ensemble (1D3Z.pdb) plus COCO-derived conformers	20	this study

U_1XQQ	DER ensemble	128	[14]

U_2NR2	MUMO ensemble	144	[15]

U_2K39	EROS ensemble	116	[10]

U_ISD	ISD ensemble (ensemble provided as an example with the ISD 1.1 package)	25	[26]

U_NNR	NOE(2)+S^2^(8)+RDC(8) restrained ensemble	32	this study

U_CNS	RECOORD ensemble calculated with CNS	25	[34]

U_CNW	RECOORD ensemble calculated with CNS in water	25	[34]

U_CYA	RECOORD ensemble calculated with CYANA	25	[34]

U_CYW	RECOORD ensemble calculated with CYANA in water	25	[34]

U_1UBQMD	5ns MD simulation started from the X-ray structure 1UBQ	32	this study

U_1G6J	Ubiquitin in reverse micelles	32	[36]

U_1V80	Ubiquitin at 30 bar	10	[37]

U_1V81	Ubiquitin at 3000 bar	10	[37]

U_2JZZ	Solid-state NMR structure	20	[38]

The ensembles were evaluated against a wide set of NMR parameters taken from the BMRB database [40] and the literature. Although for some of the ensembles specific data sets are available, we have used the parameter set valid for ambient conditions for the evaluation of each ensemble. This allowed us to characterize the differences between the ensembles in terms of their capability to reflect parameters obtained under ambient conditions.

After a literature survey, we have chosen the following NMR parameter sets, admittedly arbitrarily in some cases, for evaluating the ubiquitin ensembles:

• The initial distance restraint list was taken from the PDB database as deposited along with the structure 1D3Z[35]. From this set, all lines containing the word "or" were omitted to yield 1320 clearly unambiguous restraints used for structure evaluation.

• Backbone N-H S^2 ^values were taken from [41] (numerical data courtesy of the authors, data reported for 20°C were used).

• Cα-Hα S^2 ^order parameters are taken from [42] (BMRB entry 6466).

• N-H RDC values reported by [35] were used.

• N-Hα RDCs were taken from [43] (data set courtesy of the authors).

• Cα-Hα, C-Cα and C-Hα RDCs reported in [44] were used (data sets courtesy of the authors).

• Chemical shifts used were described in [42] (BMRB entry 6466).

• J-couplings reported in the Supplementary Table 2 of ref. [45] were used.

**Table 2:** SGCI structures used for evaluation with the CoNSEnsX approach.

Structure identifier	Description	No. of models	Reference(s)
S_1KGM	Solution NMR ensemble	10	[46]

S_COCO	Solution NMR ensemble (1KGM) plus COCO-derived conformers	20	this study

S_NN	MUMO ensemble	32	[28]

S_CNS	RECOORD ensemble calculated with CNS	25	[34]

S_CNW	RECOORD ensemble calculated with CNS in water	25	[34]

S_CYA	RECOORD ensemble calculated with CYANA	25	[34]

S_CYW	RECOORD ensemble calculated with CYANA in water	25	[34]

S_1KGMMD	5ns MD simulation started from the NMR structure 1KGM	32	this study

**Figure 3:**
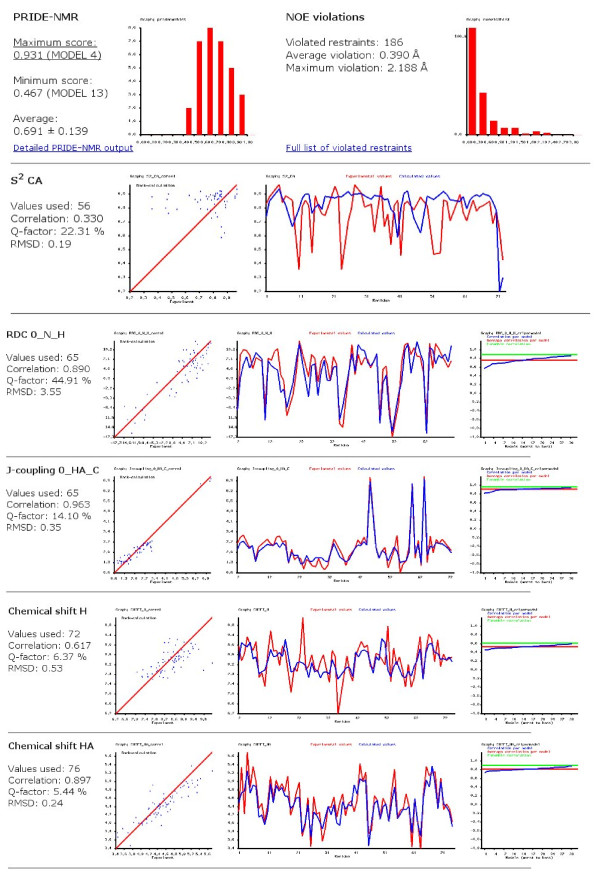
**Selected parts of the CoNSEnsX server output for the U_NNR ensemble**.

**Figure 4:**
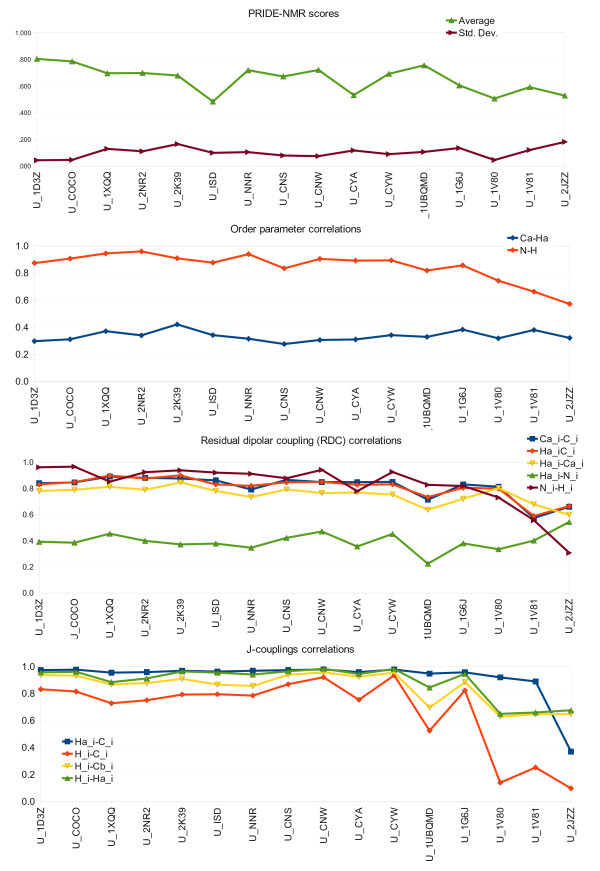
**Graphs of correlations of selected experimental parameters to those backcalculated from various ubiquitin ensembles**. Lines are shown only to make the graphs easier to read.

With the exception of ^1^H-^1^H distance restraints, all the above listed parameters were complied into a single BMRB format file that was used as input for CoNSEnsX. A sample output for the U_NNR ensemble is shown in Figure 3. Results obtained for various ubiquitin ensembles show no dramatic differences in the correspondence of structures to experimental NMR data (Figure 4). This is quite surprising at first sight given the differences in the techniques used to obtain them. We note that we have used the same dataset of experimental NMR parameters for all ensembles, thus our analysis is only relevant, at best, in assessing the compliance of structure sets determined with various methods and under different conditions to parameters in solution at ambient temperature and pressure. Put it another way, no critique of the original structure calculation approaches can be derived from non-compliance with these parameters, but the conflict of structures obtained under non-ambient conditions with the input data can indicate perceivable conformational deviation from the others.

Interestingly, all ensembles perform well in terms of amide N-H S^2 ^parameters, and poorly for Cα-Hα S^2 ^values, which are not used as restraints in any of the calculations. We note that amide N-H S^2 ^values are uniformly high except for the C-terminus, thus a protocol yielding uniformly restricted N-H vectors for all residues is still expected to give a relatively high correlation with experimental data.

Most RDC values tested yield acceptable agreement with all of the ensembles, a notable exception being the Hα-N data set. Note that RDCs were back-calculated *ab initio *using the coordinates only without applying singular value decomposition (SVD) using the experimental data. CoNSEnsX allows performing SVD by invoking PALES in best fit mode. Chemical shifts also show good agreement with experimental data for all ubiquitin ensembles, and their different sensitivity to structural factors can clearly be traced, e.g. Cβ shifts are most dependent on residue type, thus deviation could reflect assignment errors rather than being structurally relevant.

We conclude that human ubiquitin has a well-defined structure for which reliable models can be obtained by a number of different approaches, possibly reflecting the inherent overall rigidity of the structure [10]. None of the ensembles yields good agreement with Hα-Cα S^2 ^order parameters and Hα-N RDCs with first-principles approximation of the alignment tensor. Not unexpectedly, the solid-state NMR structure [38] deviates remarkably from several solution-state parameters, as can be accessed by a very low average PRIDE-NMR score. This reflects that the CoNSEnsX approach is capable of revealing structural deviations even when they are not straightforward upon visual inspection (RMSD for the 10+20-membered ensemble created by joining the U_1D3Z and U_2JZZ structures is 2.42 ± 0.7 Å). It is also apparent that only integrated investigation of multiple parameters tested is able to unambiguously reflect the deviation of the high-pressure solution structure (U_1V81) related to the experimental parameters obtained under ambient parameters. The U_NNR ensemble (Figure 2B) performs well for restrained parameters such as NOE, amide N-H S^2 ^and amide N-H RDC values and for several unused ones, like Cα and Hα chemical shifts. This is similar to the case of other dynamically restrained ensembles (U_1XQQ, U_2NR2 and U_2K39). The U_1UBQMD ensemble (Figure 2C) still yields acceptable values, although somewhat worse than the U_COCO set (Figure 2A), which shows reasonable agreement with most parameters.

### Analysis of structural ensembles of a small serine protease inhibitor

Schistocerca gregaria chymotrypsin inhibitor (SGCI) was chosen to represent small, flexible proteins in our CoNSEnsX test. There are two structural ensembles available for this molecule, one determined by 'conventional' single-conformer refinement (SCR) using X-PLOR [46] and one calculated with ensemble NOE and backbone NH S^2 ^restraining [28]. This inhibitor can be characterized by relatively low backbone Lipari-Szabo S^2 ^values around 0.7 [47], justifying its use as an example for a flexible molecule. The recently generated dynamically restrained structural ensemble is substantially more diverse and has been shown to reproduce experimental parameters better than the SCR one. SGCI is also an example of a system with limited data as only NOE distance restraints (deposited with the coordinates in the PDB), ^1^H and ^15^N chemical shifts, and backbone amide S^2 ^values are available (BMRB entry 5272 [46]).

**Figure 5:**
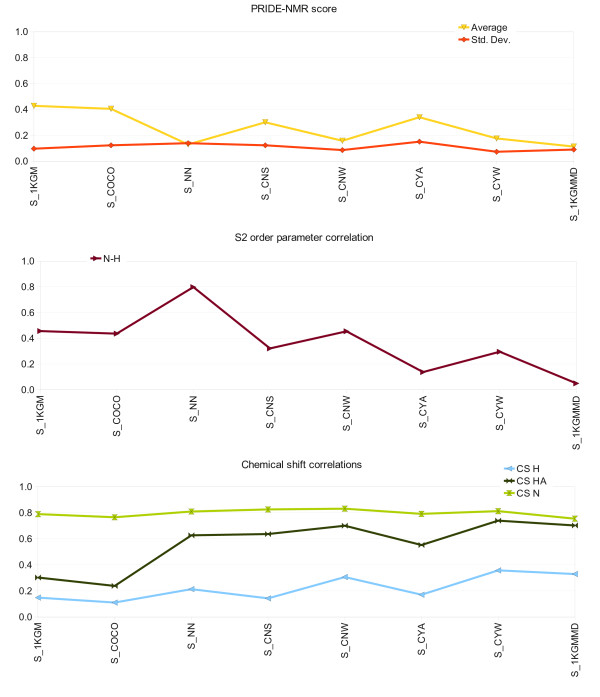
**Graphs of correlations of selected experimental parameters to those backcalculated from various SGCI ensembles**. Lines are shown only to make the graphs easier to read.

We have used various ensembles of SGCI, summarized in Table 2[28,34,46]. Among SGCI ensembles, only the dynamically restrained one reproduces experimental backbone N-H S^2 ^data. Except for the S_1KGM and S_COCO ensembles, all correspond to Hα and amide N chemical shifts reasonably well. In the SCR-derived ensemble S_1KGM, Thr9 is in a conformation which differs from all other ensembles giving rise to a back-calculated Hα chemical shift far from the experimental value. None of the ensembles tested yields acceptable correlation with back-calculated amide H chemical shifts (Figure 5).

### Analysis of structural ensembles of the intrinsically disordered γ subunit of PDE 5/6

**Figure 6:**
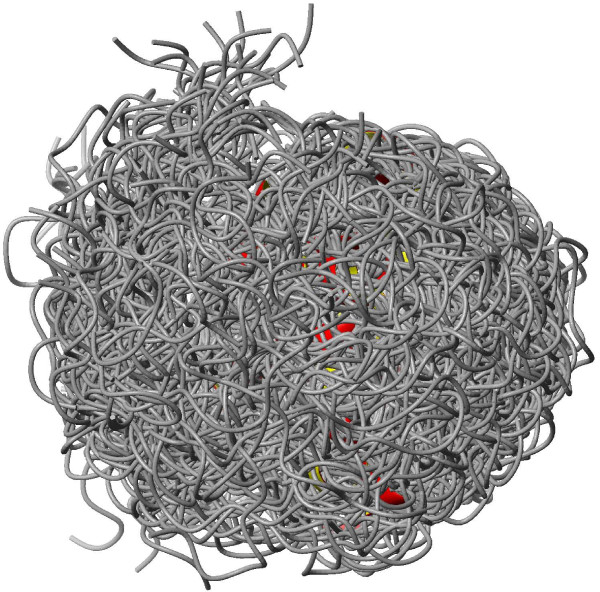
**Ribbon representation of the disordered PDE γ subunit (PDB ID **2JU4**)**. Figure prepared with MOLMOL.

**Figure 7:**
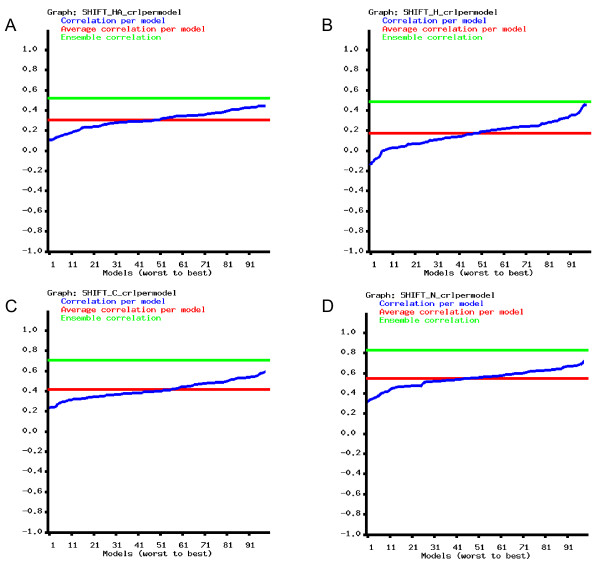
**Correlations of experimental and back-calculated chemical shifts in the 100-membered conformational ensemble of the PDE γ subunit (PDB ID **2JU4**)**. Blue line: correlation per model; red line: average correlation per model; green line: correlation calculated for the ensemble. A: Hα shifts, B: amide H shifts, C: carbonyl C shifts, D: amide N shifts.

The γ subunit of cGMP phosphodiesterase 5/6 is an intrinsically disordered protein for which a conformational ensemble is available in the PDB [48]. The 100-membered conformer set was calculated using NOE and PRE (paramagnetic relaxation enhancement)-derived restraints. The ensemble consists of highly diverse structures with a backbone RMSD over 12 Å. For this protein, only the deposited ensemble (PDB ID 2JU4[48]) was investigated. For all chemical shift types for which data are available, the correlation between experimental and back-calculated data is considerably better for the full ensemble than for any individual conformer (Figure 6 and Figure 7). This observation clearly justifies the use of such a diverse conformational ensemble for representing the conformations realized by this molecule in solution.

## Discussion

### Conformational ensembles as novel models of protein structure and their evaluation

Protein structure determination from NMR data conventionally yields a number of conformers that are all compatible with the restraints used for structure refinement. This generally reflects the uncertainty of the parameters obtained as all the conformers are compatible with them. However, the expectation that such ensembles reflect the internal dynamics of proteins is not necessarily met, one of the reasons being that one of the aims during structure refinement is to arrive at a set of structures that are reasonably similar as reflected by a low RMSD value. This can be regarded as an effort to represent solution-state protein structures as single conformers just like in X-ray crystallography - where this view would often also be an oversimplification of the observations. The uncertainty of e.g. NOE restraints can be turned to advantage by including other restraint types reporting more directly from internal dynamics to arrive at ensembles those variability primarily stems from the experimentally observed flexibility. Nevertheless, as discussed above, an ensemble of any origin might represent the internal dynamics of a protein reasonably well on a given time scale.

Ensemble-based representations can be viewed as a new type of models of protein structure incorporating the conformational diversity originating from the internal dynamics of the molecules. However, one fundamental problem resides in the wide time range of internal motions. Thus, a given structural ensemble may aim at reflecting dynamics only at a given time scale (or none). At the same time, the majority of NMR parameters measured represent the average over a number of different time scales. Thus, the expectation that a single ensemble should reflect all the motions included in the parameters might be unreasonable at present and might even be practically unachievable as it could require the use of ensembles with high number of conformers. In particular, at a given ensemble size, improving the correspondence of the ensemble to one selected parameter might only be achieved at the cost of worse compliance with several others, e.g. due to counteracting forces arising in restrained calculations [28]. The second problem is technical, meaning that typically only a small subset of all measurable parameters is available for the molecule/system studied, impairing the meaningful assessment of compliance with multiple data. Moreover, new NMR parameters can be introduced with the advancement of measurement techniques. Nevertheless, we propose that a general tool can be of help both for inspiring more measurements and the use of ensemble approaches in structure refinement.

To our knowledge, CoNSEnsX is the first structure analysis tool that handles and evaluates all input parameters in an ensemble-averaged way. This is particularly important for diverse structure sets such as those reflecting the internal dynamics of flexible molecules and ensembles of intrinsically disordered proteins. Currently, there is no consensus on the evaluation of such conformer sets [13] and it is not straightforward to coin a generally acceptable method. In these cases, individual conformers might yield substantially different results in single-conformer evaluation and structure analysis tools [49], which are clearly not suitable to offer an overall picture of the ensemble. Moreover, there are some data types, notably S^2 ^order parameters that can only be interpreted as an ensemble property. CoNSEnsX offers a simple way to assess the compliance of measured parameters with the ensemble as a whole and to judge whether the ensemble-based representation is improved relative to the single-conformer one.

### Interpreting the output of the CoNSEnsX server

The CoNSEnsX server has been specifically designed to yield information about a structural ensemble as a whole and using as many parameters as possible. Structural ensembles can be very different regarding the number and structural similarity of the conformers included. On the other hand, the availability of NMR-derived parameter sets varies from protein to protein and laboratory to laboratory. Therefore, no standardization of the output has been attempted and no arbitrary thresholds are suggested for interpreting the reported values. Rather, all data are presented to give a useful overview of the compliance to each data set. The most informative application of the CoNSEnsX approach is the comparison of multiple ensembles and evaluating their differences in reproducing different data. This is expected to facilitate both the production of ensembles meeting the goals of structural biologists more closely and choosing those that are most suitable for a particular analysis.

It must be stressed that structural ensembles might have been generated for different purposes and can contribute to the understanding of biochemical processes at different time scales. Thus, non-conformity to one or more parameter sets does not necessarily mean that the ensemble is irrelevant or unrealistic. On the other hand, it is clearly necessary to be able to judge the limitations of an ensemble-based structural representation of a particular protein.

### PRIDE-NMR as means of selecting representative conformers

As protein ensembles reflecting dynamics are substantially diverse [49], the often cumbersome task of selecting a representative conformer becomes even more difficult. It is generally expected that the selected conformer conforms to most experimental data and is in some sense an 'average structure' of the molecule. This expectation is directly opposed to the concept of representing structures with multiple conformers. Although there might not be a single 'best solution' to this problem, we suggest that the representative conformer from proteins could be selected as the one with the highest PRIDE-NMR score with the corresponding NMR distance restraint set. It should be noted that the PRIDE-NMR approach implemented in CoNSEnsX, evaluating a single NOE list against all conformers of the same protein, differs from that available in the PRIDE-NMR server. The latter is aimed at finding the most closely matching protein structure in a database to the submitted NOE list. NOE data are available for most structures to be determined by NMR, and these data represent well the fold of the protein. PRIDE-NMR is straightforward to calculate and the resulting score is an unambiguous way to assess the completeness of this representation. In addition, the distribution of PRIDE-NMR scores for individual conformers reflects the heterogeneity of the ensemble.

## Conclusions

The purpose of CoNSEnsX is to provide a quick, easy-to-use and standardized way to assess the correspondence of structural ensembles to experimental NMR data.

It is important to stress that all structures used to represent proteins, either determined from experimental information or not, are models of the actual structure, and thus can be useful for one aspect and unusable for some other [11]. Dynamically restrained ensembles represent a novel type of models, the accuracy of which needs complex and reproducible testing. CoNSEnsX offers a standardized way for this by evaluating their correspondence to a number of independent experimental data.

## Methods

### Design of the CoNSEnsX approach

The CoNSEnsX server is capable of evaluating the following types of experimental parameters using the methods listed below:

• ^1^H-^1^H distance restraints:

◦ The PRIDE-NMR approach is used to assess the correspondence of each conformer to the set of NOE restraints [31]. This means that instead of a database search as in the PRIDE-NMR server, in CoNSEnsX the submitted conformers of the same protein are compared to the restraint file. It should be noted that this makes weighting unnecessary as all investigated structures have the same length as that corresponding to the query dataset. CoNSEnsX reports the minimum, maximum, average and standard deviation of the values as well as a histogram of the distribution of the scores.

◦ Optionally, violated restraints in the ensemble are calculated using either r^-3 ^or r^-6 ^ensemble-averaging (as chosen by the user; default is r^-6^) and r^-6 ^intramolecular averaging for all ambiguous ones, e.g. for unresolved geminal ^1^H nuclei and methyl groups, etc. CoNSEnsX reports a histogram depicting the number of violated restraints vs. violation distance and a detailed list of the violations. We stress that this calculation method yields different results from standard validation tools and its results are therefore not directly comparable to those. (Table 3).

**Table 3:** Distances for two pairs of protons in the U_1D3Z ensemble of ubiquitin.

	Model									
Distance	1	2	3	4	5	6	7	8	9	10
72 HA - 42 HB1	6.35	6.33	6.32	6.21	6.30	6.37	6.36	5.97	5.93	6.42

72 HA - 42 HB2	7.73	7.72	7.87	7.59	7.62	7.83	7.77	7.40	5.87	7.91

< r^-6 ^>	*6.82*	*6.80*	*6.82*	*6.67*	*6.75*	*6.85*	*6.83*	*6.43*	*5.90*	*6.91*

	**Overall average **(< r^-6 ^>): *6.62*									

• S^2 ^order parameters are back-calculated from the ensemble as described e.g. in [17]. Importantly, the ensemble is taken as it is by the server without performing any fitting, so if the structures are not superimposed before submission, it might result in low S^2 ^values and erroneous poor correspondence to experimental ones. As it is not necessarily obvious how the molecules should be superimposed for S^2 ^recalculations, because different authors might prefer different ways, e.g. excluding highly flexible parts from the alignment, we leave this issue to the user. Currently, backbone N-H and Cα-Hα order parameters are supported.

• Chemical shifts are estimated by invoking the SHIFTX program [29] for each conformer and taking the arithmetic average of the values for each nucleus. This means that the nucleus types handled are determined the currently available version of SHIFTX, namely Cα, Hα, amide N, amide H and Cβ shifts. For glycine Hα shifts their average is used both for experimental and calculated data.

• Residual dipolar couplings (RDC) are back-calculated using the program PALES [30] for each individual conformer and then are arithmetically averaged. By default, PALES is invoked in first-principles mode as the default for the server, meaning that the alignment tensor is estimated solely based on the structure for each conformer. This also means that the alignment tensor is separately calculated for each conformer and not for the ensemble. We believe that although thus RDCs are not treated as a property of the ensemble, this type of calculation resembles the behavior of molecules in the NMR tube better, as different conformers with different overall shape might assume different orientation [50-52]. There is no restriction on the types of RDCs that can be back-calculated, as the server takes the atom pairs from the BMRB files and passes them to PALES for calculation. All calculations presented here use steric PALES, but CoNSEnsX can be easily modified to use versions considering electrostatics, if needed. The SVD mode of PALES can be turned on at the CoNSEnsX interface.

• Scalar couplings are calculated as the arithmetic average over the ensemble. For a given conformer, values are calculated from the φ backbone dihedral angle using the Karplus equation (coefficients were taken from the NMR/X-ray data rows in Table 1 of ref. [45]). Only those J-coupling types are included in the analysis for which the updated Karplus parameters are available - ^3^**J**_H_^N^_H_^α^, ^3^**J**_H_^α^_C'_, ^3^**J**_H_^N^_C_^β^, ^3^**J**_H_^N^_C_. All of these can be calculated from the φ backbone torsion angle [45].

For each type of S^2 ^order parameter, chemical shift, RDC and scalar coupling the correlation coefficient R and the ensemble-averaged *q*-factor **(Eq. 1**.) are reported:

(1) $$q=\frac{\sqrt{\sum {\left(P_{calc}-P_{\exp}\right)}^{2}}}{\sqrt{\sum P_{\exp}^{2}}}$$

*P_calc _*is the calculated ensemble-averaged parameter, *P_exp _*is the experimentally measured one, for each residue

CoNSEnsX outputs diagrams depicting the experimental vs. back-calculated values, both as a function of the sequence of the input protein. In addition, a diagram showing the correlation of each model to the given experimental parameter is returned. Histograms of the distribution of the PRIDE-NMR scores as well as the restraint violations are also reported. All recognized experimental and the corresponding calculated data are written to a text file suitable for spreadsheet handling programs for further analysis and visualization.

### Generation of protein structural ensembles used in this study

A restrained human ubiquitin ensemble (designated as 'U_NNR' for 'ubiquitin ensemble generated using NOE, NH S^2 ^and RDC data') was generated using the MUMO approach [15] implemented by our group in Gromacs 3.3.1 [53], using NOEs [35], amide N-H S^2 ^[41] and amide N-H RDC [35] restraints with force constants 105 kJ*mol^-1^*nm^-2^, 10^6 ^kJ*mol^-1 ^and 10 kJ*mol^-1^, respectively. The NOE list was purged from ambiguities by retaining (pseudo)atom pairs corresponding to the shortest distance in the minimized structure where possible, and omitting all remaining ambiguous restraints. The use of ensemble-averaged RDC restraints is available in the official Gromacs distribution [18] and can be considered as an 'extension' of the MUMO approach as described originally [15], although the term 'MUMO' itself does not refer to the restraint types used. As a starting conformer, the first model in the PDB file 1D3Z[35] was used. After minimizing to the 200 kJ*mol^-1^*nm^-1 ^force limit and addition of explicit solvent (SPC water [54]) a 1-ns position restrained simulation was run to equilibrate the system. The MUMO simulation was run for 80 ns with 8 replicas, corresponding to a total restrained simulation time of 640 ps. Conformations were sampled every 20 ps. The resulting 32-membered ensemble (Figure 2B), omitting structures at 0 ps, is designated 'U_NNR' hereafter. Conformers were fitted with MOLMOL [39] over the backbone of all residues.

Unrestrained MD ensembles of human ubiquitin and SGCI were also generated. For ubiquitin, the starting conformer was the X-ray structure (PDB ID 1UBQ[55]) available; for SGCI, we used the fifth (representative) conformer in its deposited NMR-derived structure (PDB ID 1KGM[46]). After the minimization and equilibration as described above, a single-replica molecular dynamics run was performed for 5 ns. Omitting the first 1 ns, 32 snapshots were taken by sampling the remaining 4 ns at every 125 ps, yielding the the 'U_1UBQMD' and the 'U_1KGMMD' ensembles for ubiquitin and SGCI, respectively (Figure 2C).

COCO (Complementary Coordinates) is a recently described approach that takes the protein structural ensemble as input and generates a set of conformers enriching the diversity of the input structures in a consistent manner [24]. This enlarged ensemble is expected to describe the conformational heterogeneity of the protein by generating conformers not represented in the original ensemble, but deduceable from the original ensemble coordinates. Ensembles extended with the COCO approach [24] (Figure 2A) were generated using the COCO web server [56]. For all ubiquitin and SGCI ensembles, conformers were fitted with MOLMOL [39] over the backbone of all residues.

In the coordinate file of the PDE γ subunit (PDB ID 2JU4[48]), all RCY (3-maleimido-PROXY-cysteine) residues were replaced by standard CYS (cysteine) residues before submitting the structure file to the CoNSEnsX server.

## Availability and requirements

• **Project name**: CoNSEnsX

• **Project home page**: http://consensx.chem.elte.hu

• **Operating system**: Web-based service

• **Programming language**: Perl, C++

• **Other requirements**: The server uses the SHIFTX and the PALES programs.

• **License**: Free

• **Any restrictions to use by non-academic users**: None

## Abbreviations

**BMRB**: biological magnetic resonance bank; **COCO**: complementary coordinates; **CoNSEnsX**: compliance of NMR-derived structural ensembles with experimental data; **CSA**: chemical shift anisotropy; **DER**: dynamic ensemble refinement; **EROS**: ensemble refinement with orientational restraints; **IDP**: intrinsically disordered proteins; **ISD**: inferential structure determination; **MUMO**: minimal under-restraining minimal over-restraining; **NMR**: nuclear magnetic resonance; **NNR**: ensemble calculated using NOE, amide N S^2 ^and amide N RDC data; **NOE**: nuclear Overhauser effect; **PDB**: protein data bank; **PDE**: phosphodiesterase; **PRE**: paramagnetic relaxation enhancement; **PRIDE-NMR**: probability of identity - NMR; **RDC**: residual dipolar coupling; **RECOORD**: recalculated coordinates; **RMSD**: root mean square deviation; **SCR**: single conformer refinement; **SGCI**: Schistocerca gregaria chymotrypsin inhibitor; **SPC**: single point charge; **SVD**: singular value decomposition.

## Authors' contributions

ZG and AP designed the research, BS, ZG and AFÁ wrote the CoNSensX application. All authors participated in evaluating the results and preparing the manuscript and have approved it before submission.
